# Improved Detection of Remote Homologues Using Cascade PSI-BLAST: Influence of Neighbouring Protein Families on Sequence Coverage

**DOI:** 10.1371/journal.pone.0056449

**Published:** 2013-02-20

**Authors:** Swati Kaushik, Eshita Mutt, Ajithavalli Chellappan, Sandhya Sankaran, Narayanaswamy Srinivasan, Ramanathan Sowdhamini

**Affiliations:** 1 National Centre for Biological Sciences, Tata Institute of Fundamental Research, Bangalore, Karnataka, India; 2 School of Chemical and Biotechnology, Shanmugha Arts, Science, Technology & Research Academy, Thanjavur, Tamil Nadu, India; 3 Molecular Biophysics Unit, Indian Institute of Science, Bangalore, Bangalore, India; University of Cyprus, Cyprus

## Abstract

**Background:**

Development of sensitive sequence search procedures for the detection of distant relationships between proteins at superfamily/fold level is still a big challenge. The intermediate sequence search approach is the most frequently employed manner of identifying remote homologues effectively. In this study, examination of serine proteases of prolyl oligopeptidase, rhomboid and subtilisin protein families were carried out using plant serine proteases as queries from two genomes including *A. thaliana* and *O. sativa* and 13 other families of unrelated folds to identify the distant homologues which could not be obtained using PSI-BLAST.

**Methodology/Principal Findings:**

We have proposed to start with multiple queries of classical serine protease members to identify remote homologues in families, using a rigorous approach like Cascade PSI-BLAST. We found that classical sequence based approaches, like PSI-BLAST, showed very low sequence coverage in identifying plant serine proteases. The algorithm was applied on enriched sequence database of homologous domains and we obtained overall average coverage of 88% at family, 77% at superfamily or fold level along with specificity of ∼100% and Mathew’s correlation coefficient of 0.91. Similar approach was also implemented on 13 other protein families representing every structural class in SCOP database. Further investigation with statistical tests, like jackknifing, helped us to better understand the influence of neighbouring protein families.

**Conclusions/Significance:**

Our study suggests that employment of multiple queries of a family for the Cascade PSI-BLAST searches is useful for predicting distant relationships effectively even at superfamily level. We have proposed a generalized strategy to cover all the distant members of a particular family using multiple query sequences. Our findings reveal that prior selection of sequences as query and the presence of neighbouring families can be important for covering the search space effectively in minimal computational time. This study also provides an understanding of the ‘bridging’ role of related families.

## Introduction

Proteins that belong to the same family- exemplified by significant sequence similarity - are evolutionarily related and share similar three dimensional structures and function. Two proteins are said to be remote or distant homologues, if the sequence identity among them is poor, owing to evolutionary divergence, but they share common fold and function. Detection of such distant relationships between proteins from sequence information alone, amongst a wide range of unrelated sequences having poor sequence identity, remains a challenging task. It is also crucial to understand how dissimilar sequences can adopt similar structure and function which will aid in inferring the evolutionary aspects of proteins. As the protein sequence space is very vast and is continuously expanding, as compared to structural space, detecting such distant relationships is still a pivotal task in the field of computational biology. Considerable efforts are required to establish distant relationships amongst proteins.

**Figure 1:**
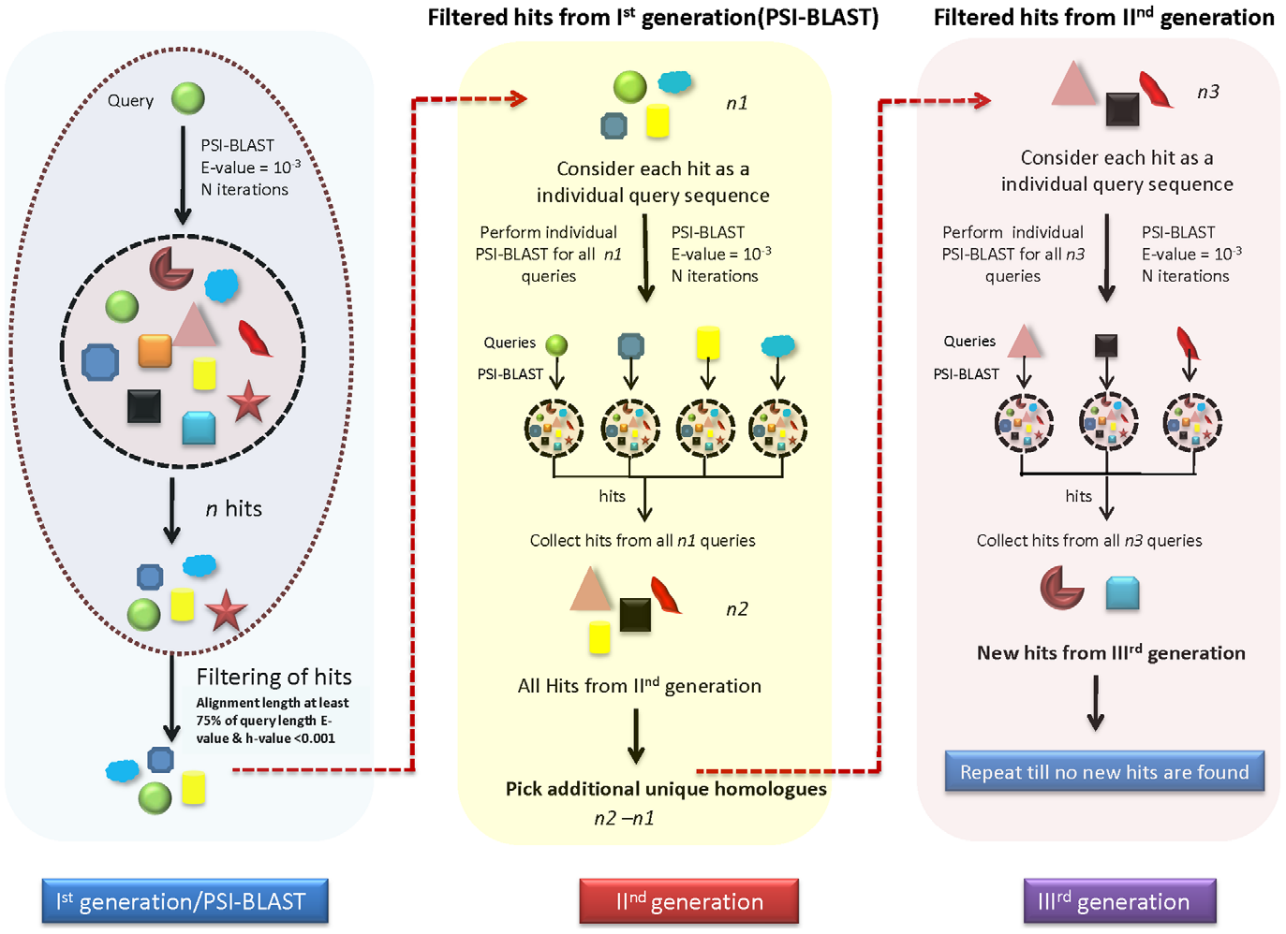
Schematic comparison of PSI-BLAST and Cascade PSI-BLAST. Database of sequences is represented as a dashed line circle having many sequences (represented with different colors and shapes), green sphere represents query sequence. Dashed oval represents PSI-BLAST, while transparent blocks indicate three different generations of Cascade PSI-BLAST. First generation of Cascade PSI-BLAST is equivalent to PSI-BLAST but contains filtered hits. These filtered hits are propagated to further generations in Cascade PSI-BLAST. New hits from each generation are considered as a seed sequence for next generation.

In order to detect such relationships amongst proteins, many search methods can be employed like sequence to sequence, sequence to profile and profile to profile comparisons. Sequence-based approaches basically employ HMMs [1], [2], profiles [3], [4], templates [5], intermediate sequences [6]–[8] and machine-learning tools [9], [10] to detect true relationships. Structure-based methods perform relatively better as structures are conserved better than sequences [11]. Some of the methods like consensus-based approaches have been found to be highly successful as evident in recent editions of the CASP (Critical Assessment of Techniques for protein Structure Prediction) experiment [12], [13], where accuracy had increased when different methods were combined to generate a consensus [14]. Although many of these methods are very powerful in homology detection, yet the coverage of complete sequence space is not guaranteed. If the relationship between two sequences cannot be detected directly due to poor sequence identity, presence of a third sequence which is homologous to both can be used to establish relationship between the other sequences. Use of such intermediate sequences that share sequence features of more than one protein is found to be effective in detecting distant protein similarities [6], [15]. Intermediate sequences are likely to populate the sequence space and relate sequences which are difficult to connect by normal search procedures. Such an approach has been shown to provide 70% of improvement over direct sequence comparison methods [6], [16]. Several algorithms have been proposed to find the distant members from which the most widely used is a well-known method called PSI-BLAST. Even though PSI-BLAST approach is sensitive to detect remote homologues, due to rapidly increasing sequence space, a complete coverage is not possible. An alternative approach called Cascade PSI-BLAST, which involves use of intermediate sequence connections to perform recursive PSI-BLAST searches (starting from each of the homologous hits obtained at the end of the previous run (‘generation’)), is proved to be more effective [4]. In this method, all the homologous sequences recognized in one ‘generation’ were further subjected to PSI-BLAST using stringent thresholds and any additional homologues, not found previously were also included (Figure 1). The Cascade PSI-BLAST search propagation is performed at least till the ‘third generation’ to cover most of the distant homologues of a protein family. Such non-directed search propagation maximizes the chances of detecting distant homologues by effectively scanning the protein “fold space”.

In this study, remote homology relationships have been analyzed using Cascade PSI-BLAST method in three plant protein families: prolyl oligopeptidases, rhomboids and subtilisins. In all the three families, significant sequence dispersion exists. We have tested how the coverage of sequence searches varies when different protein family members (including those whose structures are not yet determined) are presented as a query. Different query sequences are expected to accumulate their own distant members. We have focused on systematically analyzing the role of neighbouring families in making distant connections using the statistical approach of jack-knifing. This analysis will significantly help in understanding the influence of neighbouring families on a query family in identifying its related homologues. By understanding such contributions, remote homology searches can be further improvised to cover huge protein sequence space with high efficiency and accuracy.

Plant serine proteases have been shown to be involved in diverse processes regulating plant development and defense response [17]–[20]. Recent sequence analysis on plant serine proteases showed a detailed comparison of *A. thaliana* and *O. sativa* for all the superfamilies of serine proteases [21]. Prolyl oligopeptidases (POP) are serine proteases belonging to α/β hydrolase fold; they retain a catalytic triad of Ser554, Asp640 and His681 as active site residues which lie between α/β hydrolase and β-propeller domains of POPs [22]. POPs have been implicated in the degradation of biologically important peptides such as peptide hormones and various neuropeptides [23]. Rhomboids are intra-membrane serine proteases that cleave transmembrane helices, to liberate proteins that participate in important processes, such as cell signaling and gene regulation [24], [25]. Unlike the usual catalytic triads in serine proteases, they are known to have a catalytic dyad (Ser201, His254) in their active site [26]. This enzyme has one domain consisting of six transmembrane helices and five loops, in which loop1 includes WR motif, is important for activity [27]. Subtilisins are one of the most extensively studied proteins and are classified as peptidase S8, having highly conserved catalytic triad (Asp137, His168, Ser325) but in a different order [28]. Large number of subtilases in *A. thaliana* and *O. sativa* may be due to multiple duplication events [29]. Structural determination reveals that it belongs to subtilisin-like fold, which has eight parallel and one antiparallel β-strand forming a twisted β-sheet which is surrounded by α-helices on both sides [30].

In this work, we have shown the use of cascaded sequence searches and multiple queries to improve sequence coverage. Difference in coverage is observed for different query sequences, which implies that choosing the best query before searching for the remote homologues can be an important step. This can even reduce the computational time of rigorous searches and also increases the accuracy of prediction. Coverage analysis was performed for all the queries and an appreciable increase in the coverage of plant serine proteases at family, superfamily and fold level was noticed. To show wide applicability of this approach, similar analysis was also carried out on 13 families covering all the structural classes of SCOP (Structural Classification of Proteins [31]) database to understand the impact of multiple queries of a family on coverage. Cascade PSI-BLAST performed better at all the above levels as compared to a single generation of PSI-BLAST.

## Methods

### Generation of Augmented Database

All sequence searches were performed using PALI (Phylogeny and Alignment of homologous protein structures) database which is an information resource of proteins of known three dimensional structures and their homologous sequences [32]. Earlier rigorous analysis on serine proteases by Tripathi and Sowdhamini [21] had reported a number of genes encoding POP, rhomboid, subtilisin-like proteins in genomes of *A. thaliana* and *O. sativa* as shown in Table 1 and Table S1. All these protein sequences were downloaded from the TAIR [33] and Rice genome annotation project [34], and were appended to PALI database for generation of ‘augmented PALI-plus’ database which includes PALI database of homologous sequences and plant members of the particular family (POP/rhomboid/subtilisin, depending on the sequence search type) used in this analysis (see Figure S1 for details). As the number of expected relationships is known *a priori,* this augmentation of plant serine protease sequences to PALI-plus database has been performed for assistance in the assessment of Cascade PSI-BLAST approach. Each entry in the PALI-plus database have been annotated with the corresponding SCOP code to facilitate its tracing, which allows easy identification of true members of the particular fold/family (Table 2).

**Table 1:** Number of genes encoding POP, rhomboids and subtilisin-like proteins in genomes of *A. thaliana* and *O. sativa* (Tripathi and Sowdhamini, 2006) [21].

Protein Family	*A. thaliana*	*O. sativa*
**Prolyl oligopeptidase**	23	23
**Rhomboids**	20	18
**Subtilisins**	56	58

**Table 2:** Statistics of PALI-plus database.

Plant Family	No. of sequences atfamily level	No. of sequences atsuperfamily level	No. of sequences atfold level
**Prolyl oligopeptidase**	138 (c.69.1.4)	9528 (c.69.1)	9528 (c.69)
**Rhomboids**	38 (f.51.1.1)	209 (f.51.1)	209 (f.51)
**Subtilisins**	616 (c.41.1.1)	850 (c.41.1)	850 (c.41)

### Selection of Domain of Interest

All queries were scanned against HMMPFAM (using HMMER3.0 [1]) and NCBI-CDD [35] to retrieve the catalytic domains of plant sequences of POP and subtilisins since they retain multi-domain architectures. Matching of domain boundaries was also carried out by aligning the structural members present in Protein Data Bank (PDB) [36] with each of the queries. Selected domain was further used for the Cascade PSI-BLAST searches.

### Search Method: Cascade PSI-BLAST

Cascade PSI-BLAST approach was used to detect the distant relationships in the three serine protease families. This approach used the strengths of multiple generations of PSI-BLAST to detect distant homologues. All the resulting hits were considered individually as queries and again the search was employed to identify homologues which have not been identified earlier, thereby performing cascaded search for three generations. Cascade PSI-BLAST searches were employed using the standalone version of the program with all the plant protein sequences as queries for the analysis. Calculation of coverage was then carried out at the levels of family, superfamily and fold. Examination of false positives and true positives were performed at all the above three levels. A hit was considered as a false positive if it did not belong to the same fold as that of the query. Finally, coverage gathered by PSI-BLAST was compared with that for all the three generations for evaluating the sensitivity of this approach.

### Cascade PSI-BLAST Using Multiple Queries

Instead of using any one random query from a protein family, all the plant serine protease sequences of a particular family were used as queries to understand the role/importance of different sequences in enhancing the coverage at family, superfamily and fold level. For other SCOP classes, ten queries of a family were selected randomly for performing sequence searches.

### Parameter Selection

All the runs were carried out by considering E-value and H-value (inclusion threshold for building PSSM) of 10^−3^ for all three generations. Since the shorter alignments might be erroneous hits, only the hits having alignment length greater than 75% of the query length were taken. This percentage is referred as ‘length-filter’ or ‘query coverage filter’. All the queries were propagated at least till third generation to facilitate the detection of all the new homologues.

### Jackknifing of Families

In statistical terms, the Jackknife estimator is about systematically re-computing the statistical estimate, leaving out one or more observations at a time from the sample set. This approach has been applied here to the protein superfamily by removing one family (all protein sequences within that family) at a time from it, and observing the difference in coverage of the remote homologues. In order to understand the effect of individual families in covering the remote homologues, highly populated superfamilies should be chosen so that the elimination of neighbouring families will be robust. For this purpose, α/β hydrolase and TIM folds were selected due to their diverse composition of superfamilies and families. In TIM fold, we chose metallo-dependent hydrolase superfamily, a popular entity of this fold, which had about 18 families within, while in α/β hydrolase fold, α/β hydrolase superfamily was selected.

### Performance Measures

For all the queries, true positives, true negatives, false positives and false negatives were identified for all the three generations. Further, coverage, sensitivity specificity and precision score were also calculated. Mathew’s correlation coefficient [37] measure was also computed to confirm the quality of prediction. Here, coverage was defined as total number of true associations identified by sequence searches (at family/superfamily/fold level) by total number of sequences present in database of that family/superfamily/fold. False positives at family/superfamily/fold level are defined as hits which do not belong to same family, superfamily and fold respectively as query sequence.
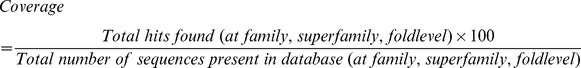


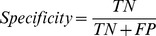


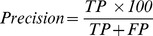


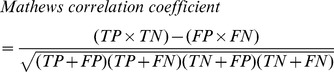
where, TP  =  Number of true positives, FP  =  Number of false positives, TN  =  number of true negatives and FN  =  number of false negatives.

## Results and Discussion

Plant members of all the three serine protease families were considered as queries to understand the behavior and contribution of each sequence in accumulating more homologues during Cascade PSI-BLAST searches. Stringent E-values and length filters were employed to minimize the number of false positives. Sequence searches were performed until three generations to accumulate distantly related protein sequences of a particular family. It has already been reported that searches up to three generations detect most homologues of the query [4]. At each generation, significant numbers of hits were used as queries for further exploration in the sequence space. If the hits obtained did not belong to the same fold by PALI definitions, those hits were considered as false positives. False positives and true positives were monitored for all the generations starting from multiple queries. Coverage was assessed by examining the number of hits belonging to the same fold, namely true positives, in comparison to the total number of expected true positives.

### Detection of Homologues Increases with Cascade PSI-BLAST

Detection of remote homologues within the family was performed at two levels: firstly, within the plant sequences which were appended into PALI-plus database and secondly, for sequences of a particular family present originally in PALI-plus database. Cascade PSI-BLAST searches were performed on rhomboid and subtilisin serine protease families.

#### Rhomboid family

**Figure 2:**
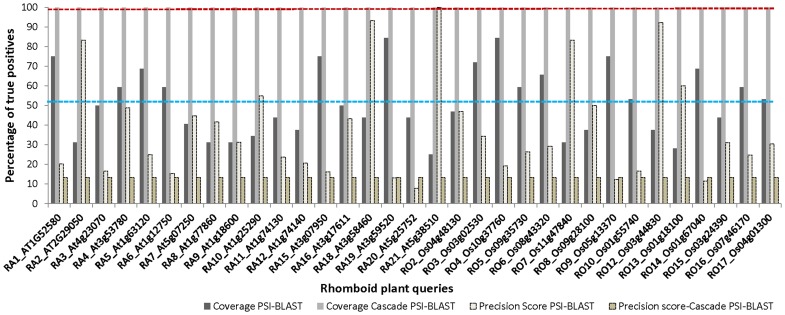
Coverage of plant rhomboids at family level (only plant rhomboids are considered). Black bars indicate coverage of PSI-BLAST, while grey bars indicate Cascade PSI-BLAST. Average coverage of PSI-BLAST and Cascade PSI-BLAST are indicated by blue and maroon dashed lines. White and dark yellow dashed bars indicate precision score of PSI-BLAST and Cascade PSI-BLAST.

An unrooted phylogenetic tree was computed by the neighbour-joining method [21] starting from multiple sequence alignments of rhomboid protease domains from *A. thaliana* and *O. sativa*. 38 rhomboid proteases were distributed into seven clades. Clade-I, having largest number of queries (15 members), was used initially for this analysis. Cascade PSI-BLAST on above queries of clade-I, against the corresponding augmented PALI-plus database, gave rise to almost full coverage. As expected, queries were able to identify other members from the same clade very efficiently, and in order to investigate its sensitivity for cross-clade associations, searches were performed on all the plant members of all the seven clades of rhomboids (38 sequences). Results for all 38 sequences were analysed and no false connections were noticed. This might point to the closely–knit single-membered rhomboid-like superfamily, whose members have sequence identities ranging from 39–69%. While the graphs (Figure 2 and S2) suggested that most of queries registered 97% coverage with precision score of 100 (Table S2) by using Cascade PSI-BLAST, on an average, 86% (SD = 0.10%, N = 34) of plant homologues could be obtained using this approach at the family level, while ∼42% could be identified using PSI-BLAST. For the PALI-plus sequences, coverage was 87% (SD = 0.14%, N = 34) which was better than through PSI-BLAST (24% coverage). The coverage figures remained the same at superfamily and fold levels, as there is only one family in rhomboid-like superfamily (Figure S2). Accuracy levels were found to be quite high with 89% sensitivity, ∼100% specificity and a MCC of 0.9 when searched through Cascade PSI-BLAST.

In spite of the high coverage obtained using Cascade PSI-BLAST, we noticed five queries (two from *O. sativa* (RO1, RO11: Table S1) and three from *A. thaliana* (RA13, RA14, RA17: Table S1) showed very poor coverage. Detailed examination showed that these queries were shorter than others and contain smaller number of transmembrane helices than the normal six transmembrane helices found in rhomboids (results obtained from HMMTOP, [38] data not shown). Alignment with structural entry (PDB id: 2IRV) showed absence of any active site conservation or WR motif conservation in these sequences (Figure S3). Their annotations were examined using Pfam [39] sequence search and Gene Ontology server [40]. Except one query (RO1: Table S1), the sequences were associated with Der1-like family or UBA/TS domain. However, in Pfam, Der-1 like family is clustered with rhomboids in the same clan and may be very distantly related. Henceforth, those queries were not used for further analysis. For RO1 query (Table S1), the length-filter parameter was relaxed to 30% (from the default 75%) and this led to higher coverage (26% to 61% at family level and from 0 to 99% at superfamily and fold level) with no false positives. Length-filter relaxations, when performed on other four low-coverage queries, sometimes led to no increase in coverage or even led to the accumulation of many false positives. The accuracy measures for rhomboid family (excluding the four low-coverage non-rhomboid queries: RA13, RA14, RA17, RO11 in Table S1) reach up to ∼100% of sensitivity, specificity and MCC of one.

#### Subtilisin family

When homology searches were applied on proteins of the subtilisin family, 20% coverage could be obtained (average coverage at all levels) using PSI-BLAST, while using the Cascade PSI-BLAST approach, all homologues which were present within PALI-plus database were covered. At the family level, use of PSI-BLAST gave rise to 44% coverage, while using Cascade PSI-BLAST ∼100% (SD = 0.74%, N = 114) coverage could be obtained for all the plant homologues. The use of PSI-BLAST covered only 9% homologues from the entire PALI-plus sequences (at family level), while all homologues could be identified using Cascade PSI-BLAST approach. This trend was also present at superfamily (and fold) level, where only 8% coverage was obtained using PSI-BLAST, whereas 99% (SD = 1.67%, N = 114) coverage could be obtained using Cascade PSI-BLAST (Figure S4). This particular result proved very promising, as our objective of finding distant homologues at the fold level was possible by Cascade PSI-BLAST. Accuracy measurements (by standard methods) revealed that searches using Cascade PSI-BLAST (in case of subtilisins) retain 99% sensitivity and specificity, with MCC of 0.78. Hence, from the case studies of rhomboid and subtilisins, use of Cascade PSI-BLAST approach was more effective than PSI-BLAST for the detection of distantly related sequences.

### Multiple Queries are Better than Single Representatives for Sequence Searches

**Figure 3:**
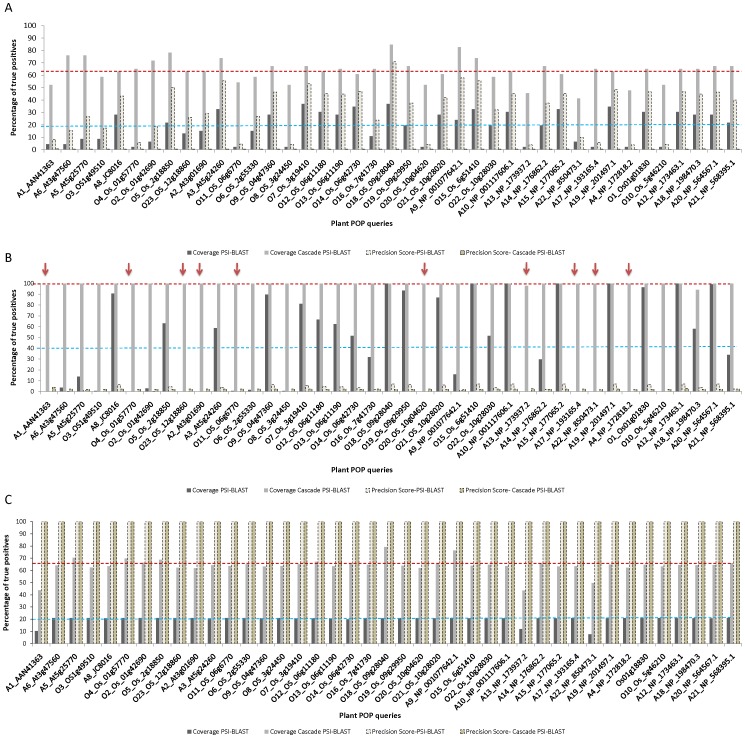
Coverage of plant POPs a) family level (only 46 plant POPs are considered) b) family level –non plant POPs, present in PALI-plus c) superfamily/fold level. Average coverage of PSI-BLAST and Cascade PSI-BLAST are indicated by blue and maroon dashed lines. Arrows indicates queries where PSI-BLAST could not pick any member but coverage of Cascade PSI-BLAST was 100%. White and dark yellow dashed bars indicate precision score of PSI-BLAST and Cascade PSI-BLAST.

Use of multiple sequence representatives has already been reported in the literature to improve coverage [41] and it has been considered as a highly effective procedure to accumulate more remote homologues than using a single sequence as a representative. Earlier studies on Cascade PSI-BLAST were carried out using only a single structural representative sequence from SCOP database [4]. Therefore, the calculated coverage may have always been biased towards a particular sequence. Here, an attempt was made to understand the role of individual queries in increasing the number of remote homologues of that family. For this purpose, prolyl oligopeptidase family was selected since it belonged to a highly diverse α/β hydrolase fold having 41 different families. Performance of each plant POP sequence was examined to understand the importance of each sequence in accumulating additional members. Figure 3 shows the performance of each query in terms of coverage at family and fold level. During first generation, average coverage was found to be only ∼19% (SD = 2.8%, N = 41), which then increased to ∼64% (SD = 6.19%, N = 41), when queries were propagated to second and third generations of searches. Figure 3a clearly shows how different queries give rise to different coverage in identifying remotely related members.

Searches starting from some of the queries gave rise to high coverage (more than 80% for O18 and A9 in Table S1), while for few other queries (A13, A22: Table S1), the search direction gave rise to a coverage of just 40%. We observed that differences in coverage at family level were also reflected at fold level as shown in Figure 3c. Most of the approaches to detect distant homologues lack high coverage at superfamily level, mainly because of the poor sequence identities between the members of the same superfamily. However, sequence searches using Cascade PSI-BLAST approach gave rise to good coverage at superfamily and fold level as well. Analysis of coverage at family level sequences (which were already present in PALI-plus database) was found to be very interesting, as these sequences have high (>30%) average sequence identity. So, it was expected that PSI-BLAST could retain coverage close to 100%. However, the overall PSI-BLAST coverage was 45% only, whereas propagating these searches further (Cascade PSI-BLAST) gave rise to full coverage (SD = 0.96%, N = 41). Some of the sequences were found to be very interesting (arrows shown in Figure 3b), as PSI-BLAST failed to identify the related members, whereas using Cascade PSI-BLAST, all other related sequences could be identified which might act as bridging points connecting the query to its own family members.

### Impact of Co-existing Domains

**Figure 4:**
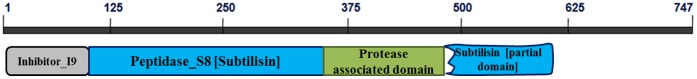
Domain architecture of subtilisin domain in plants (NCBI-CDD).

Multi-domain proteins have evolved as a result of duplication and combination of domains. Studies have also found that they are more prominent in eukaryotes (60%) while archae and bacteria have only 40% of multi-domain proteins [42]. The analysis of domain architectures also highlighted the fact that a power law is followed for nearby domains, in which there are a small number of large families with many types of neighbours, and a large number of families with few types of neighbours [43]. The functionality of such multi-domain proteins was determined by its domain composition and its interactions with other domain families [44]. We encountered such an example in our study of subtilisins which shows the impact of such domains, especially the insert domains [45]. Initially in our study of subtilisins, Cascade PSI-BLAST was performed on full-length queries of 56 *A. thaliana* queries. A simple PSI-BLAST run achieved 86% coverage in identifying plant subtilisins at the family level (86% coverage), while 96% coverage could be achieved with Cascade PSI-BLAST. However, while searching for PALI-plus sequences at the family level, only 3% coverage could be achieved using PSI-BLAST, compared to 92% coverage using Cascade PSI-BLAST (SD = 0.74%, N = 114). A similar trend was also seen at superfamily and fold level, whereas PSI-BLAST run gave rise to 12% coverage, 70% coverage was possible with Cascade PSI-BLAST (SD = 1.67%, N = 114). Although the results were encouraging, these were accompanied by the presence of false positives. They were found to be from very diverse folds (Galactose-binding domain like, PA domain, EGF-type module and CUB-like fold) in spite of “query filter” check and stringent-length filter (see Methods for definitions). Upon performing a traceback of the false positives, it was found that such connections emerged mostly from two particular regions in the query. The queries were then checked for their domain architecture and regions responsible for most of false positives were found to be from the co-existing domains, PA (protease-associated) domain and Inhibitor_I9 domain (Pfam nomenclature) (Figure 4 and Figure S5). The Inhibitor_I9 domain, found at N-terminal of subtilisin, is generally removed prior to activation of the enzyme, and these subtilisin pro-peptides were known to function as molecular chaperones by assisting in folding of mature peptidase, but have also been shown to act as “temporary inhibitor”. The PA domain has been found as an insert in the diverse proteases and formed a lid-like structure that covered the active site in active proteases, and was also involved in protein recognition in vacuolar sorting receptor [46], [47]. Hence, as remarked by Park *et al.* [48], intermediates arising from multi-domain proteins during “intermediate sequence search” approach could introduce errors and thus may have been responsible for this upsurge in false positives (Figure S6).

**Figure 5:**
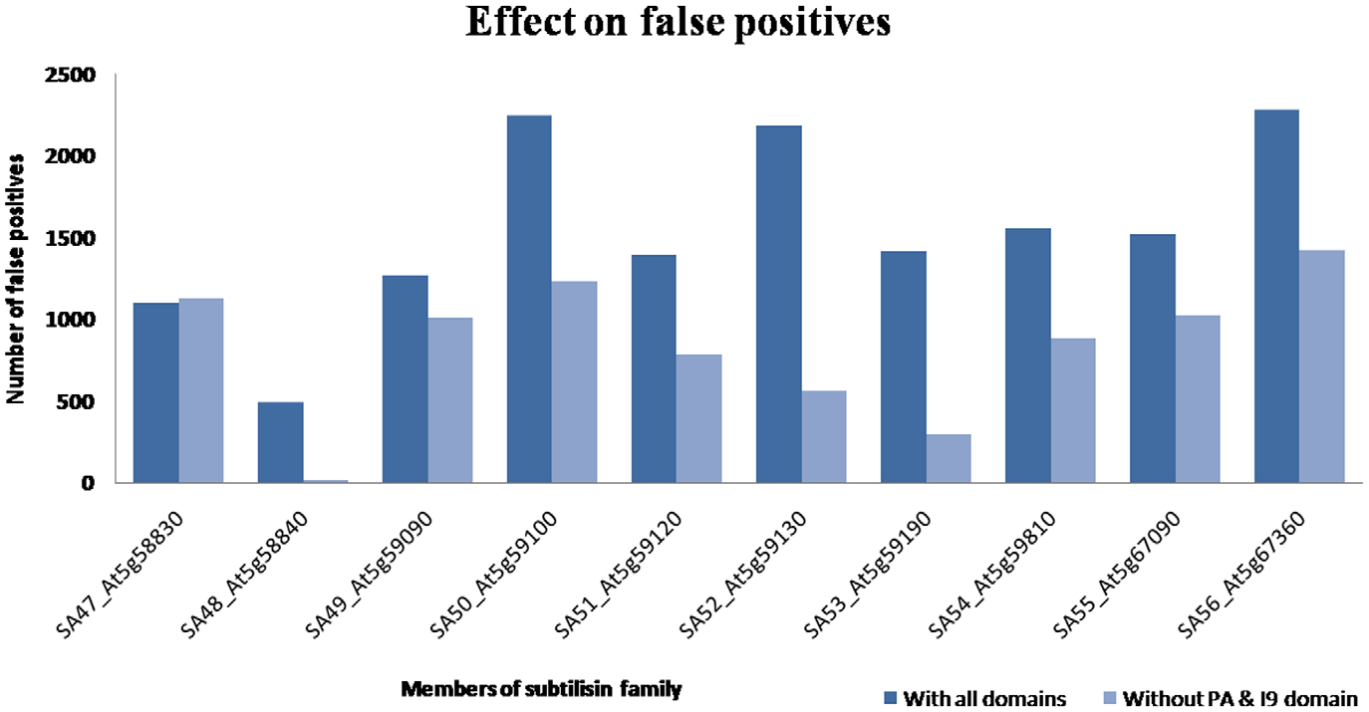
Impact of co-existing domains on false positives.

Therefore, accurate boundaries of subtilisin domains (after removal of PA and Inhibitor_I9 domains), as detected by NCBI-CDD server (conserved domain database), was used to make single domain queries and Cascade PSI-BLAST run was performed on all of them again. This resulted in the dramatic decrease of number of false positives (Figure 5). The remaining false positives could be identified due to relaxed length filters at the end of third generation.

### Connections of Homologues in Different Generations

**Figure 6:**
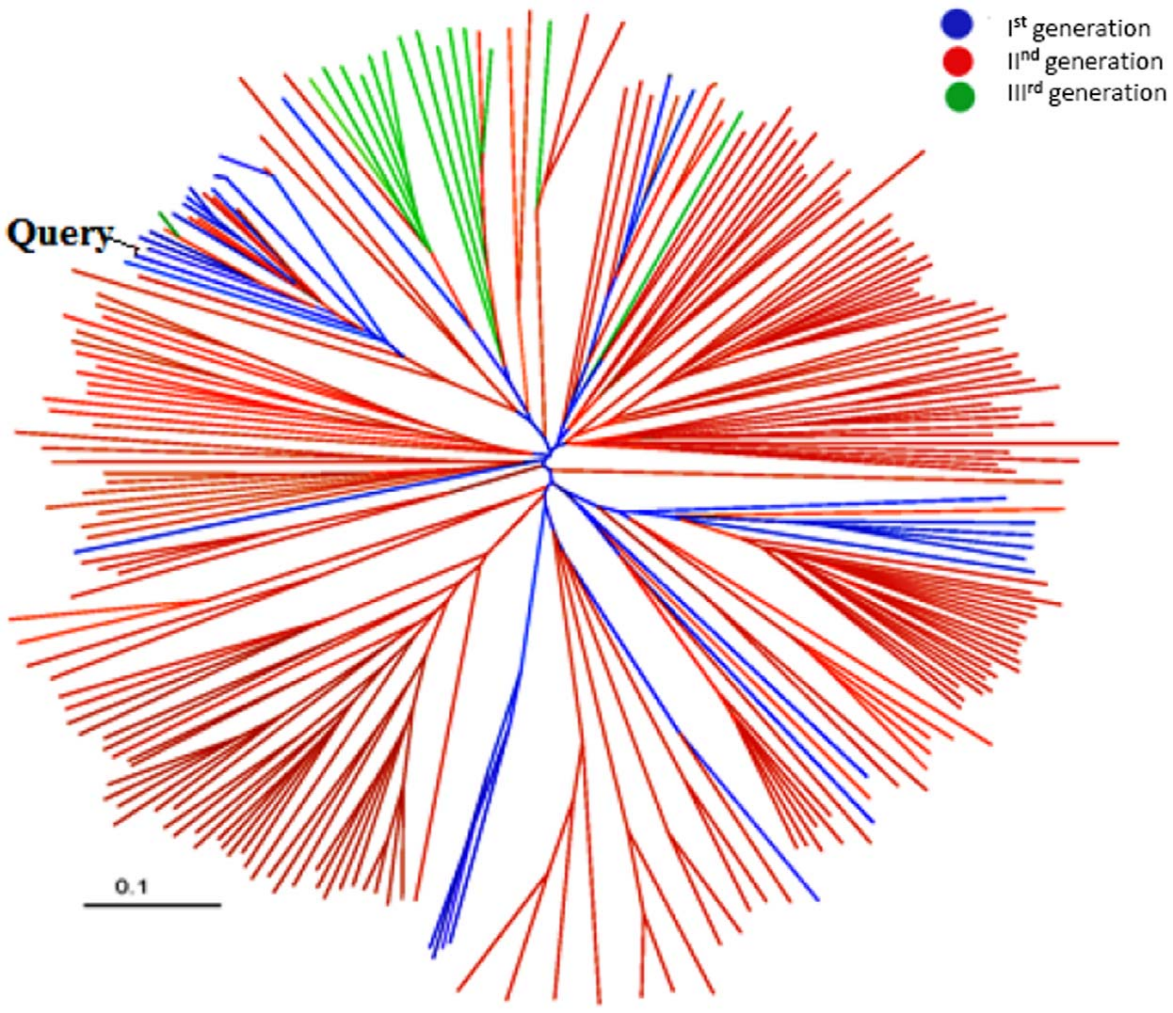
Phylogenetic tree of rhomboid query with its homologues. Query is shown in black and the homologues, obtained in different generations in Cascade PSI-BLAST, are shown in different colors. Generation I, generation II and generation III are shown in blue, red and green color respectively.

**Figure 7:**
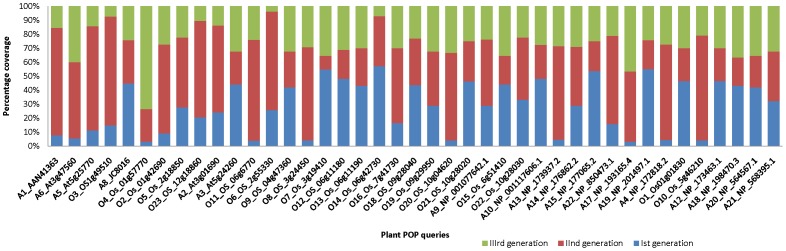
Coverage statistics for different plant POPs as queries, observed at the family level. True positives obtained through first generation are indicated by blue color, second generation (red) and third generation (green).

One of the main strengths of Cascade PSI-BLAST approach is the use of intermediates for querying further in the sequence space to collect distant relatives. Here, each generation of sequence search plays an indispensable role in identifying its nearest members. To understand the role of intermediates, rhomboid family was examined in detail. Contribution of each of three generations in the accumulation of homologues was checked and the maximum number of homologues were identified in the second generation. In order to assess the connections between the homologues collected, their phylogenetic clustering was performed and coloured generation-wise for one of the queries (RO2) of rhomboids as shown in Figure 6. Plant members of rhomboids are quite dispersed and formed separate clusters. Intermediates (of second generation) are shown in red color, which completely fills the gap between first (blue) and third generation (green). In most of cases, homologues identified in the second generation were widely spread out, wheras those identified in the third generation was found to be tightly clustered. Figure 7 also shows the importance of intermediates in covering sequence space in POP family. Here, for few of the queries, first generation hits are very low (A1, A17, O10), but presence of intermediates enables the identification of the other entries.

### Cross-family Connections

**Figure 8:**
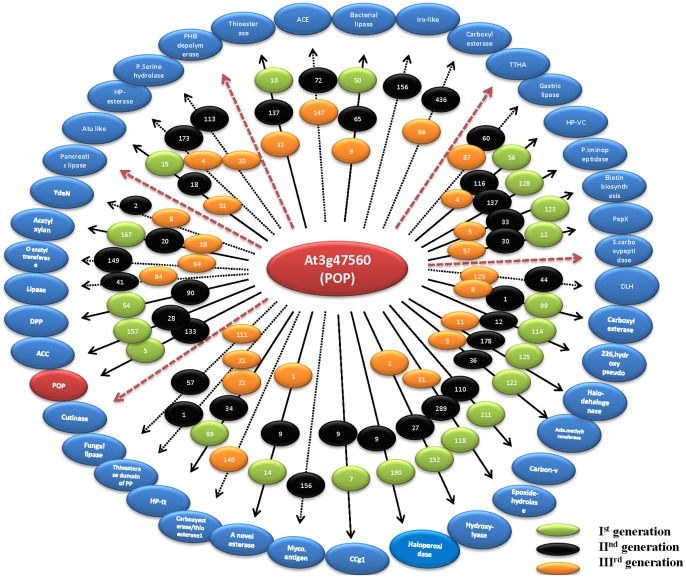
Cross-connection between families within a single fold (α/β hydrolase fold). One of POP sequence (At3g47560) is used as a query. Blue ellipse indicates α/β hydrolase families, green, black and orange ellipse indicates number of true positives found at first, second and third generation of Cascade PSI-BLAST respectively. Dashed arrow indicates families where first generation could not pick any member, but second and third generations have picked. Maroon arrows indicate families where no true positives could be found in three generations.

Cascade PSI-BLAST approach involves implementation of intermediates which enables in bridging gaps in the sequence space and as the investigation was carried out at the fold level, it can be hypothesized that the “bridging” intermediates can be found in other families present in the same fold apart from the family of our interest. We next performed a detailed examination of cross-family connections within a fold. In this respect, α/β hydrolase fold was selected due to the presence of diverse and large number of families within the fold. Figure 8 clearly shows how different sequences were identified during three different generations for one of the PoP queries (A6-At3g47560). In the first generation, few of the families were identified; this number was further increased during second and third generations.

Intriguingly, some of the families like carboxyesterases, acetyl xylan, acyl amino acid releasing enzymes and mycobacterial antigens helped in accumulating more members of POP family in further generations. For example, POP query picked 99 members from carboxyesterase family, which when further propagated, identified 10 more members from same family in further generations (Figure 8). To understand this in detail, a clustering diagram of structural members of 41 families was obtained (Figure S7), which was followed by calculation of average pairwise sequence identity with POP structural member. All the families were divided according to sequence identity with POP into three bins: sequence identity >20%, sequence identity <15% and between 15–20%. Interestingly, accumulation of more homologues was not based on sequence identity of query (POP) with other families. For instance, mycobacterial antigen family retains less than 15% sequence identity with POP, but this sequence enabled efficient identification of nearest neighbours. This analysis showed how intermediates of different families enabled cross-talks across families, which could turn out to be beneficial in accumulating more homologues of the query belonging to the same family.

### Implementation of Multiple Queries on other SCOP Classes

SCOP database provides detailed information about the evolutionary relationships of known proteins on the basis of structure and function. Determining such protein relationships using computer algorithms is very helpful in assigning functions to hypothetical proteins and those whose structures are not yet determined. Therefore, to test the wide applicability of our findings, the above analysis was also carried out on random families chosen from all the classes of SCOP database. Some of highly populated superfamilies were selected from SCOP database and comparison of coverage of PSI-BLAST and Cascade PSI-BLAST was performed (using single query sequence) (Table S3). We found that cascaded search could cover 22%, 31% and 27% more average coverage at family, superfamily and fold level respectively, for diverse protein superfamilies, with an average precision score of 71% which is very assuring. By cascading PSI-BLAST searches on multiple protein families of unrelated folds, 36% increase in coverage at the family level was observed, along with a 43% decrease in precision score (please see Table ST3). At the superfamily level, there is an observed 58% increase in coverage and 23% decrease in precision score. At the fold level, however, there is a 65% increase in coverage by cascading the sequence searches accompanied by 26% decrease in precision score. These observations clearly reveal that there is a trade-off between coverage and precision score in applying sensitive sequence search algorithms. At higher levels of structural hierarchy, the extent of ‘loss’ in precision score is fairly minimal by the cascaded approach, suggesting that there is little chance that the specificity is lost in making connections at the fold level.

Two highly populated folds were chosen from each class, from which ten queries of each family were selected at random to perform coverage analysis of each query sequence at family, superfamily and fold level (Table S4). We observed that with Cascade PSI-BLAST there was appreciable increase in coverage with precision score 81%. Use of multiple queries of a family was found to be promising as different queries and displayed differences in finding its own family members. Numbers of remote connections were also dependent on type of query used, as some queries picked members from other families while others did not. These results show a wide applicability of our approaches.

### Jackknifing of Families

The jackknifing studies (please see Methods for definition) were carried out on α/β hydrolase and metallo-dependent hydrolase superfamily. Before proceeding for Cascade PSI-BLAST runs with jackknifing, clustering analysis of α/β hydrolase and metallo-dependent hydrolase superfamily was carried out to identify the query families *i.e.* families whose sequences can be considered as a query. We found that POP in α/β hydrolase and dihydroorotase in metallo-dependent hydrolase superfamilies are suitable query members, since the position of these families were found to be in the centre of the phylogenetic tree. This suggests that they can enable recognition of large number of homologues from all the families in their respective superfamily. In each of the runs, one family of the superfamily was removed from the PALI-plus database and was searched against the query. The query was kept the same for all the jackknifing runs for a particular superfamily, so that the coverage results obtained after the removal of various families can be compared. The sequences from all the generations were checked at family, superfamily and fold levels.

**Figure 9:**
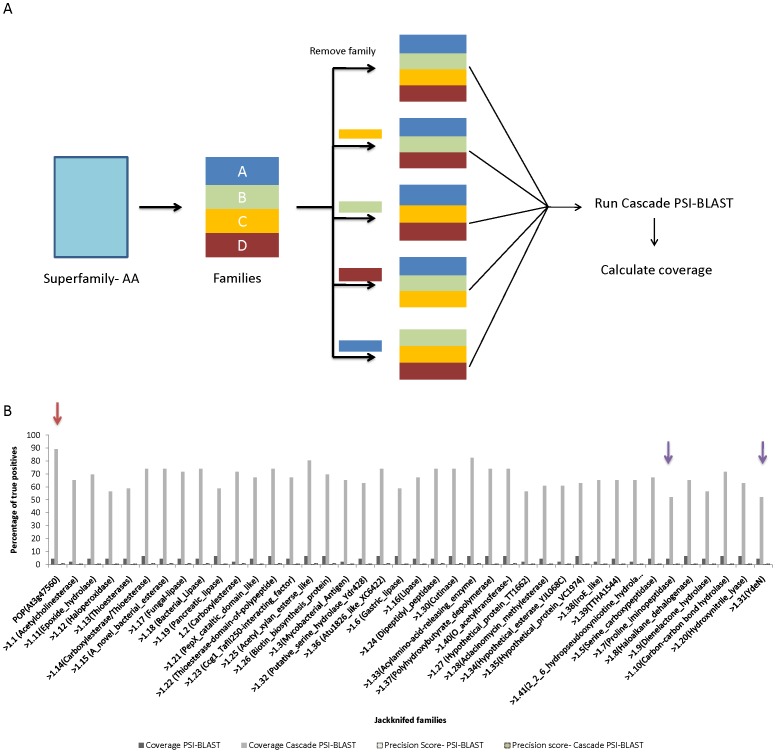
Jackknifing of the α/β hydrolase superfamily using POP as a query sequence. A) Schematic representation of jackknifing runs. B) Coverage at the POP-family level. Red arrow indicates that all the families were included in the Cascade runs. Blue arrow indicates the families where coverage had dropped. White and dark yellow dashed bars indicate precision score of PSI-BLAST and Cascade PSI-BLAST.

Jackknifing of the α/β hydrolase superfamily showed interesting trends, as we noticed that inclusion of all the families could cover 90% of the members, while removal of the families like proline-iminopeptidase, YdeN reduced the coverage to 52% as shown in Figure 9. Removal of nearest members of POP family like ACC (acyl amino-acid releasing enzyme), DPP (dipeptidyl peptidase) reduced the coverage to 82 and 74%, respectively. Similarly, with inclusion of all the families, the fold level coverage was 65%, while removal of family carboxyesterase and acetylcholinesterase reduced it by 5% (Table S5). We also noticed that difference in coverage after such systematic removal of families was higher, when Cascade PSI-BLAST was used but it was found to be negligible while using PSI-BLAST. This depicts the importance of other families during second and third generations of Cascade PSI-BLAST.

Similarly, Cascade PSI-BLAST runs were carried out for metallo-dependent hydrolase superfamily having 18 families. Coverage analysis at the family level shows that all the query-family members could be identified using PSI-BLAST in all the runs, irrespective of whether the other family is present or absent in the database. Searches using Cascade PSI-BLAST also showed a similar statistics (Figure S8). In order to understand the remote homologue coverage here, the study was progressed to the analysis at the superfamily level. Cascade PSI-BLAST approach of sequence searches brought about an appreciable increase in the coverage of all superfamily members. For example, when the family c.1.9.9 (SAH/MTA deaminase) was removed, the coverage dropped from 99% to 91%, *albeit* considerably greater than the normal PSI-BLAST coverage of 25%. The intermediate sequences obtained in the first generation enables the identification of other distantly related sequences in the same superfamily. This interconnectivity between the families was analyzed.

The fold coverage increased considerably when c.1.9.2 (alpha subunit of urease, catalytic domain) was removed. A potential reason for this might be that the family was involved in a ‘profile-trap’ [4]. Sequence dispersion may have caused some family to find homologues within their superfamily repeatedly and hence was not able to cover other different families with the same fold even after three generations. Fold coverage statistics, as of three generations, explains that when some more generations are analyzed, it is possible to bring in all the remote homologues that form the TIM-barrel fold.

### Conclusions

The intermediate sequence search approach, employed in Cascade PSI-BLAST, maximizes the identification of homologues in a manner more rigorous than the earlier approaches. It increases the potential of detection of distant relationships between proteins by propagating the searches through each intermediate and the results highlight the effective increase in the coverage at family, superfamily and fold level. In families like rhomboids and subtilisins, this has enabled 100% coverage. Cascade implementation also increases the sensitivity, specificity, precision score and MCC of remote homologue detection by exploring the entire sequence search space using multiple queries. Across the families analyzed in this study, Cascade PSI-BLAST protocol effectively enables the detection of distant relationships at the superfamily level. The protocol is also efficient at the fold level, for instance, in subtilisins it is able to increase the average coverage up to 70% (SD = 1.67%, N = 114), whereas sequence search using a single generation of PSI-BLAST can glean up to 12% (SD = 15.39%, N = 114) only.

Previously, efforts have been made using a single query as a starting point for searching homologues [4]. However, use of multiple queries proved to be more effective than dependence on a single sequence as a starting point [41]. It enhances the accuracy of prediction and potency of finding distant relationships across different folds. This approach was implemented on a diverse α/β hydrolase fold having 41 different families and was observed that some of the sequences were more efficacious than others in establishing distant connections through sequence searches. This suggests that multiple members of a family are useful to recognize distant members of diverse families/folds like α/β hydrolase or TIM.

Using this approach, we have also highlighted the inter-dependence of different family members of same superfamily/fold. Many cross-family connections were examined, which showed the importance of an individual sequence in accumulation of distant members of its own. Whereas it is possible to employ Cascade PSI-BLAST to connect the sequence space by identifying homologues, its specificity is highest at the fold level. For instance, as seen in highly-populated α/β hydrolase superfamily/fold, Cascade PSI-BLAST helps to assimilate all the possible homologues, which could not have been picked by direct connection with the query. Although this protocol was very effective in accumulating distant members at the fold level, maximum coverage was not reached, which highlights the fact that this is an open area for further research. We believe this study will be instrumental in guiding the search for remote homologs by advising against use of multiple domains or insert domains during the search as it may lead to false positives.

Further, we have also attempted to understand the influence of families in α/β hydrolase and metallo-dependent hydrolase superfamily, using systematic removal of families to understand the influence of other families of the same superfamily in improving coverage. Implementation of cascade searches on different SCOP classes revealed the generic nature of this approach which can be used on any SCOP family to identify distantly related members. Benchmarking approach, using a structurally annotated database like PALI-plus, has enabled tracking true positives which are otherwise difficult to find using non-redundant sequence database due to possible lack of annotations. By this study, we propose a generalized protocol for identifying remote homologs which includes using multiple queries as starting point followed by cascade searches of selected sequences for at least three generations to cover the fold space. These searches can be performed against any protein sequence database like NR and SwissProt and should prove beneficial in understanding and discovering remote homologs with better accuracy.

## Supporting Information

Figure S1 **Schematic of construction of dataset.**
(TIF)

FigureS2 **Coverage of rhomboids present in PALI-plus database at the fold level.** White and dark yellow dashed bars indicate precision score of PSI-BLAST and Cascade PSI-BLAST.
(TIF)

Figure S3 **Conservation of active site and WR motif in rhomboid family illustrated in the multiple sequence alignment performed by PRALINE-TM.**
(TIF)

Figure S4 **Coverage of plant subtilisins present in PALI-plus at the family level (same is in superfamily/fold level) at a) for plant subtilisins only and b) for non-plant subtilisins c) at fold level.** White and dark yellow dashed bars indicate precision score of PSI-BLAST and Cascade PSI-BLAST.
(TIF)

Figure S5 **Alignment of a false positive with inhibitor_I9 domain in the subtilisin family.**
(TIF)

Figure S6 **Trace-back of false positives to show high structural similarity causing influx of false positives even after excising out the co-existing domains in subtilisin.**
(TIF)

Figure S7 **Clustering diagram of α/β hydrolase superfamily members.**
(TIF)

Figure S8 **Jackknifing of metallo-dependent hydrolase superfamily using dihydroorotase as a query sequence.** a) Coverage at the dihydroorotase family level b) Coverage at the superfamily level and c) Coverage at fold level. White and dark yellow dashed bars indicate precision score of PSI-BLAST and Cascade PSI-BLAST.
(TIF)

Table S1 **Locus identifiers of genes encoding POP-like, rhomboid-like and subtilisin-like plant serine proteases.**
(DOC)

Table S2 **Precision score of POP, rhomboids and subtilisin query sequences.**
(DOC)

Table S3 **Coverage comparison of PSI-BLAST and Cascade PSI-BLAST of large superfamilies of SCOP database.**
(XLS)

Table S4 **Implementation of multiple sequence queries of a family using Cascade PSI-BLAST on different SCOP classes.**
(XLS)

Table S5 **Jackknifing of the α/β hydrolase superfamily using POP as a query sequence representing coverage at the fold level.**
(DOC)
