# Supplementary figures and images for: Improved Detection of Remote Homologues Using Cascade PSI-BLAST: Influence of Neighbouring Protein Families on Sequence Coverage
Source: PLoS One. 2013 Feb 20;8(2):e56449. doi: 10.1371/journal.pone.0056449 (PMC3577913; doi:10.1371/journal.pone.0056449)

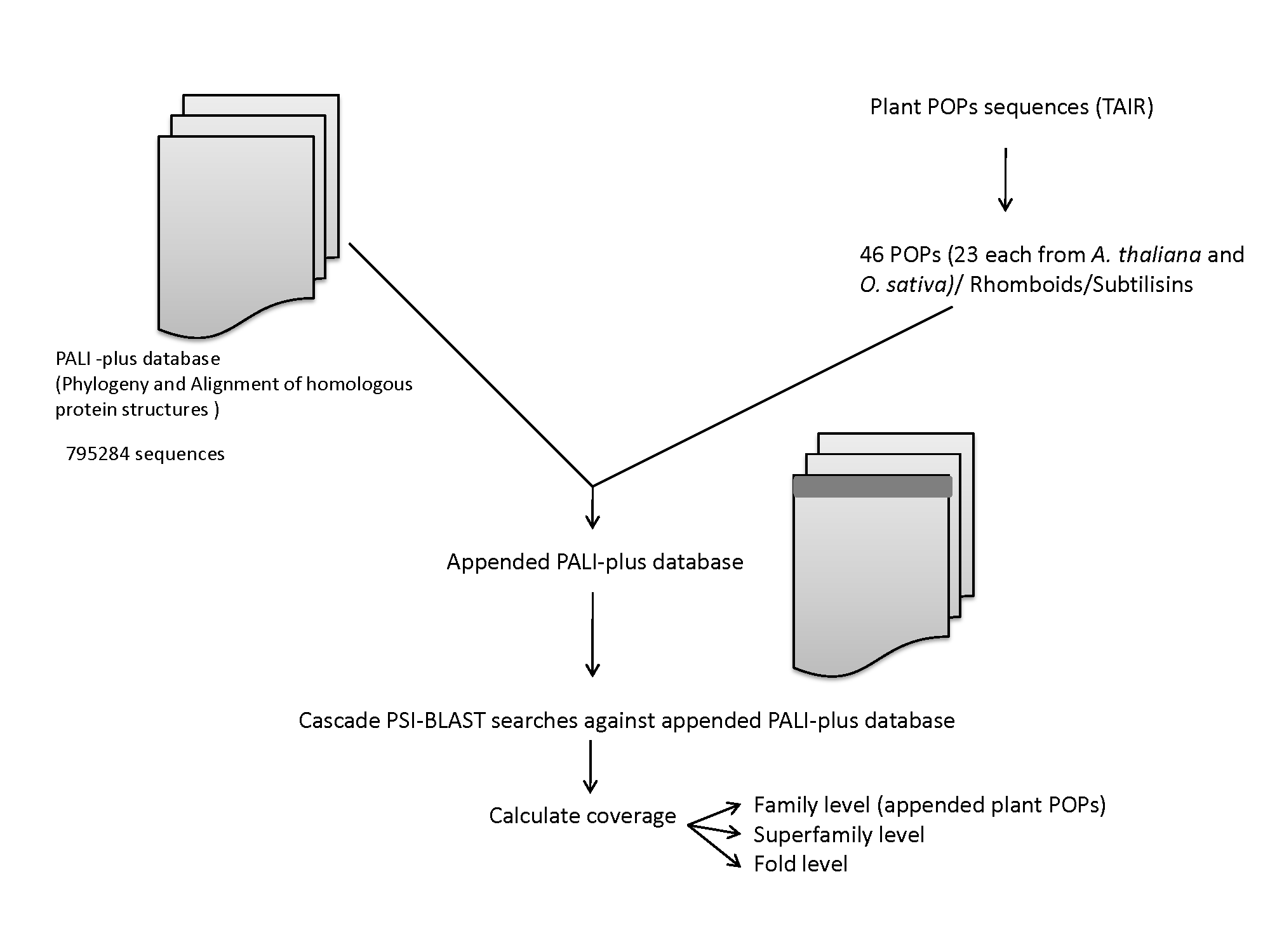

Supplement: Figure S1 — Schematic of construction of dataset. (TIF) [file pone.0056449.s001.tif]

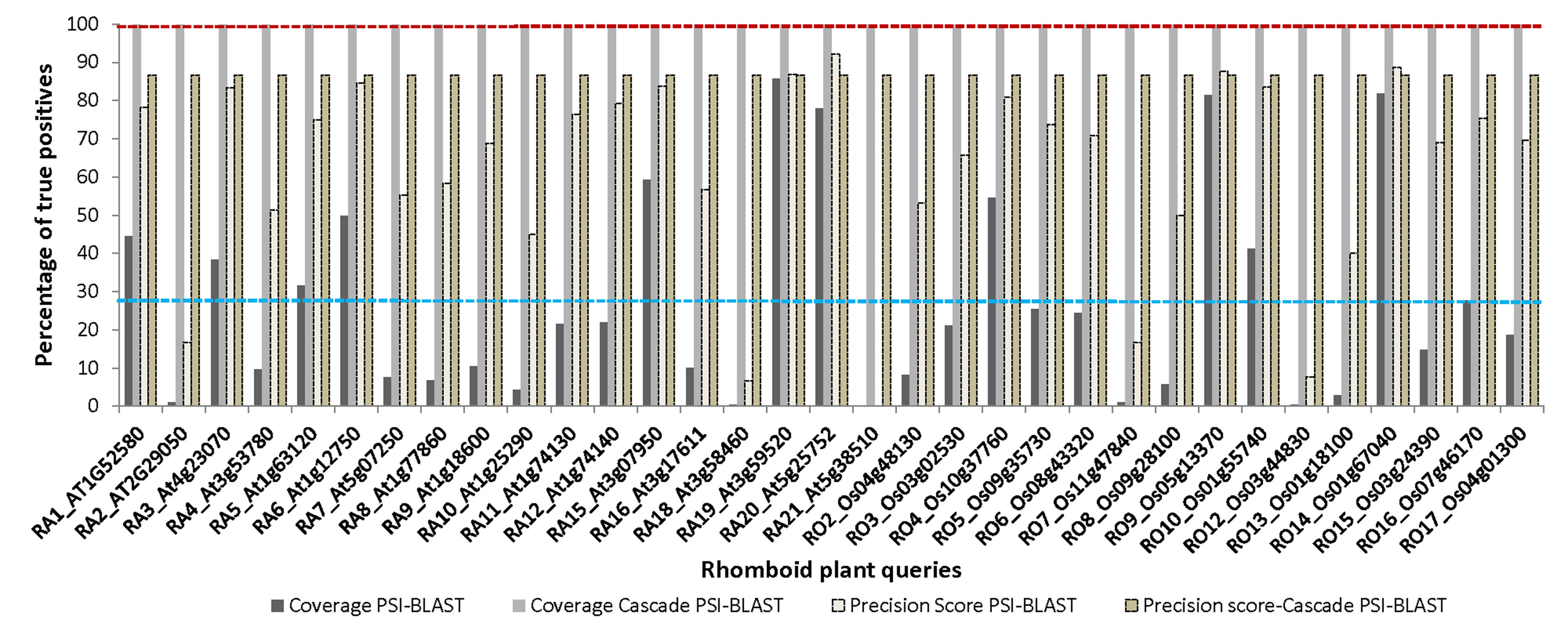

Supplement: FigureS2 — Coverage of rhomboids present in PALI-plus database at the fold level. White and dark yellow dashed bars indicate precision score of PSI-BLAST and Cascade PSI-BLAST. (TIF) [file pone.0056449.s002.tif]

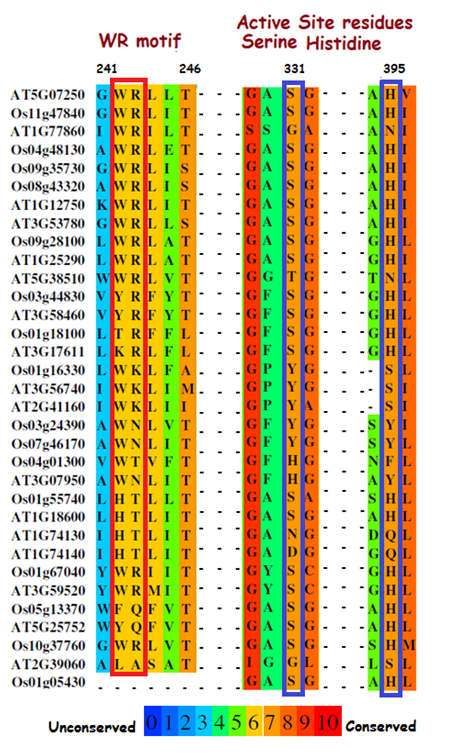

Supplement: Figure S3 — Conservation of active site and WR motif in rhomboid family illustrated in the multiple sequence alignment performed by PRALINE-TM. (TIF) [file pone.0056449.s003.tif]

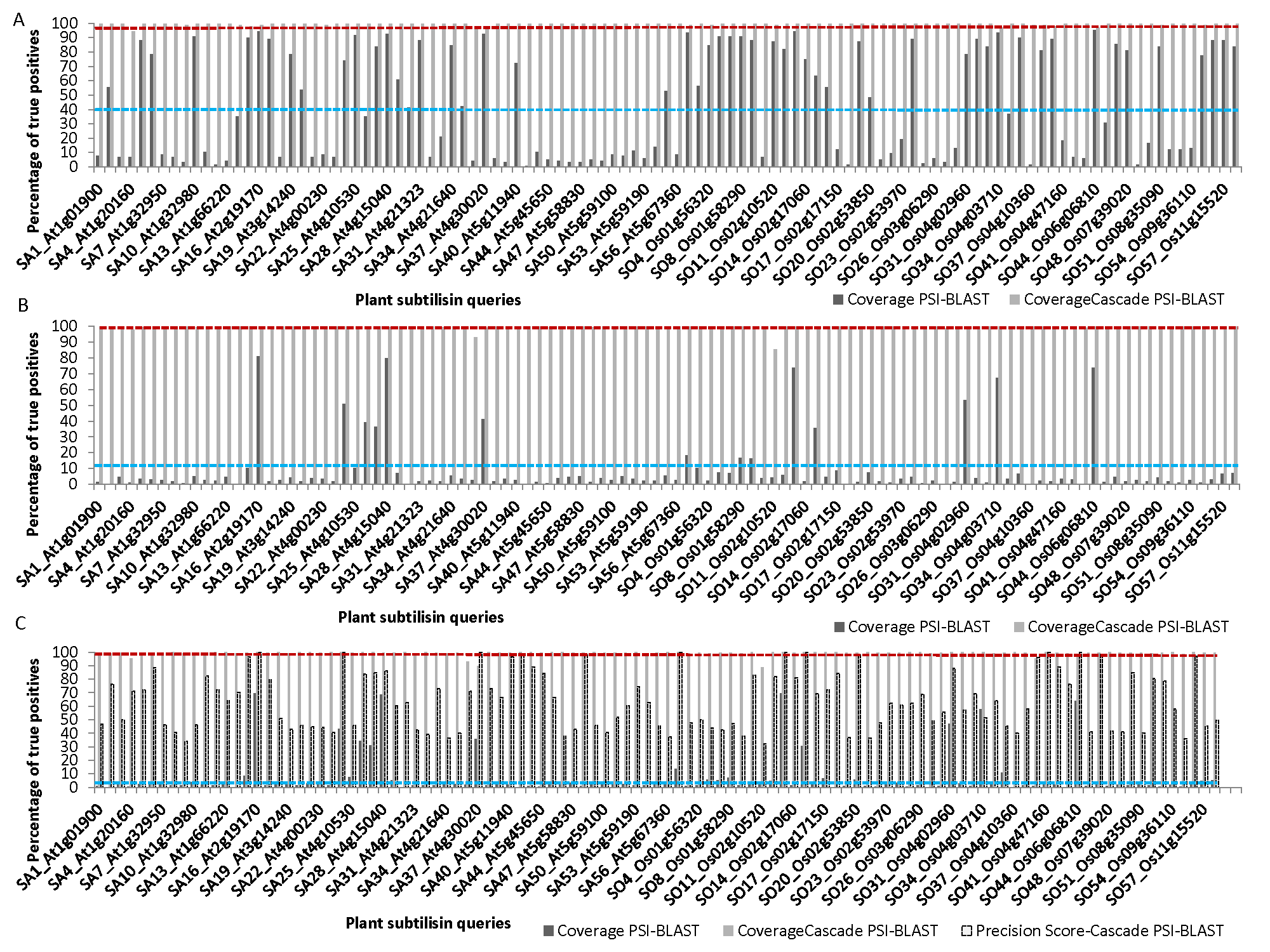

Supplement: Figure S4 — Coverage of plant subtilisins present in PALI-plus at the family level (same is in superfamily/fold level) at a) for plant subtilisins only and b) for non-plant subtilisins c) at fold level. White and dark yellow dashed bars indicate precision score of PSI-BLAST and Cascade PSI-BLAST. (TIF) [file pone.0056449.s004.tif]

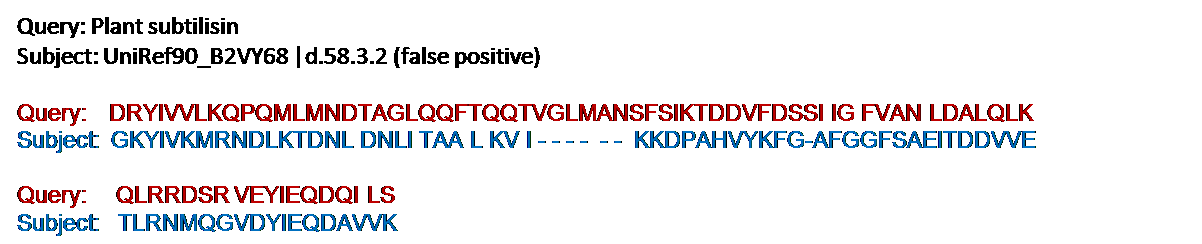

Supplement: Figure S5 — Alignment of a false positive with inhibitor_I9 domain in the subtilisin family. (TIF) [file pone.0056449.s005.tif]

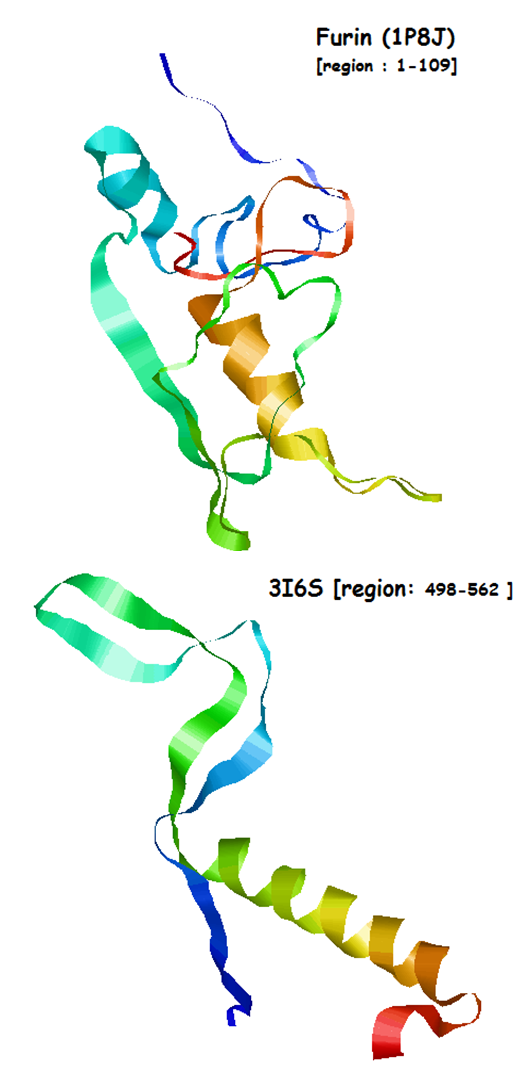

Supplement: Figure S6 — Trace-back of false positives to show high structural similarity causing influx of false positives even after excising out the co-existing domains in subtilisin. (TIF) [file pone.0056449.s006.tif]

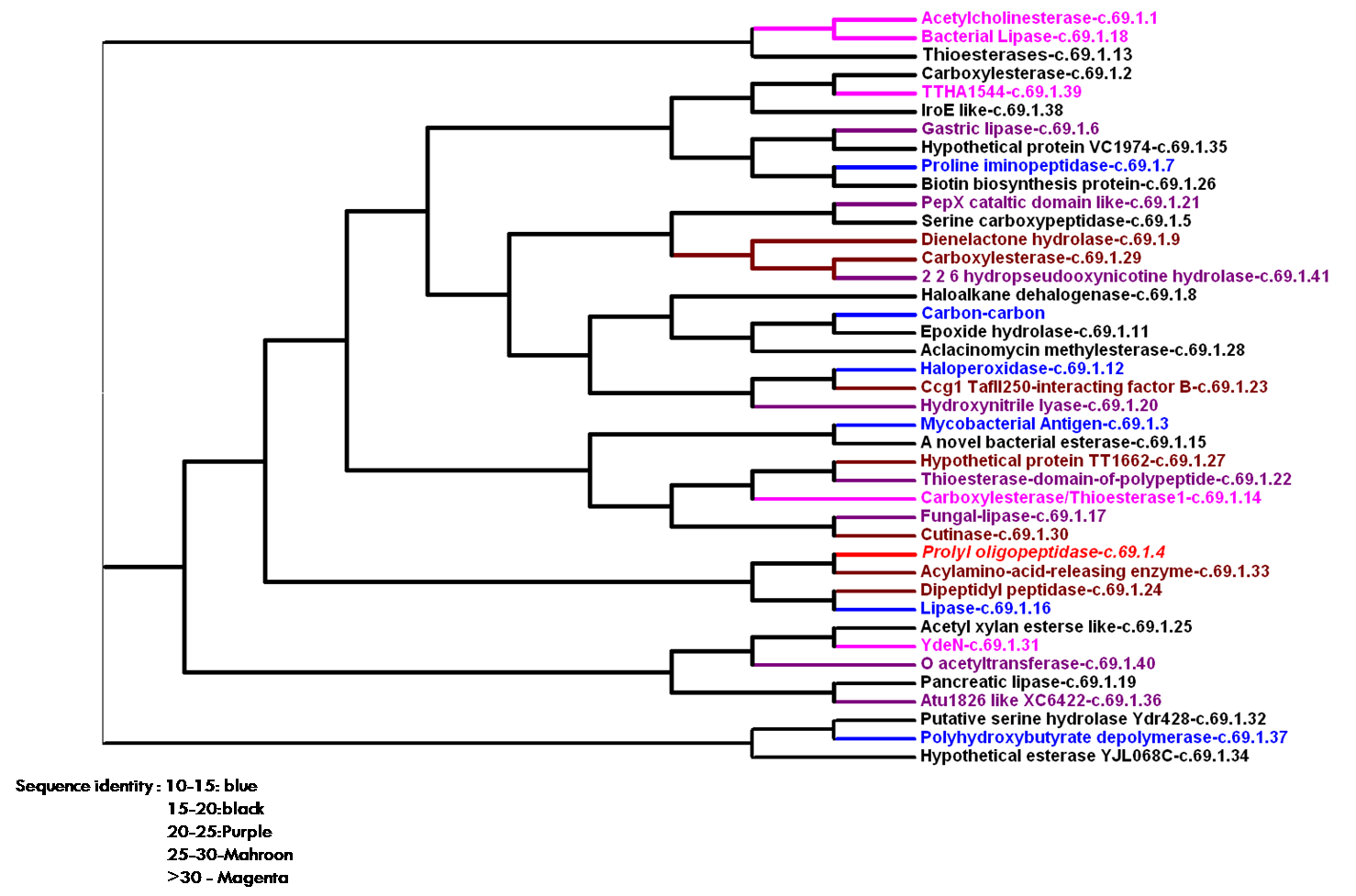

Supplement: Figure S7 — Clustering diagram of α/β hydrolase superfamily members. (TIF) [file pone.0056449.s007.tif]

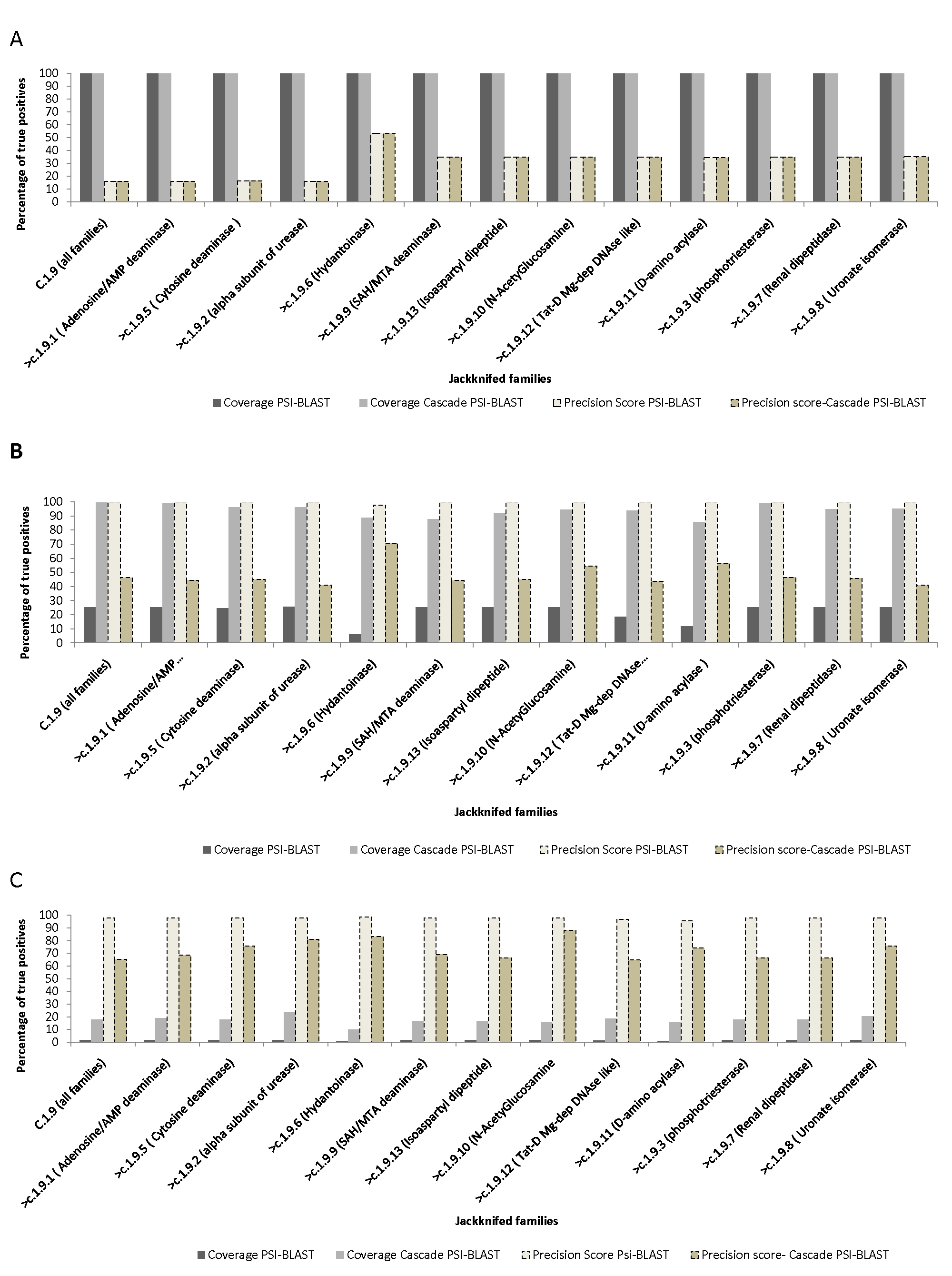

Supplement: Figure S8 — Jackknifing of metallo-dependent hydrolase superfamily using dihydroorotase as a query sequence. a) Coverage at the dihydroorotase family level b) Coverage at the superfamily level and c) Coverage at fold level. White and dark yellow dashed bars indicate precision score of PSI-BLAST and Cascade PSI-BLAST. (TIF) [file pone.0056449.s008.tif]
