# Supplementary material for: Improved Detection of Remote Homologues Using Cascade PSI-BLAST: Influence of Neighbouring Protein Families on Sequence Coverage
Source: PLoS One. 2013 Feb 20;8(2):e56449. doi: 10.1371/journal.pone.0056449 (PMC3577913; doi:10.1371/journal.pone.0056449)
Supplement: Table S1 — Locus identifiers of genes encoding POP-like, rhomboid-like and subtilisin-like plant serine proteases. (DOC) [file pone.0056449.s009.doc]

Table S1: Table shows locus identifiers of three families.

| Locus Identifier | |
| --- | --- |
| **Prolyl oligopeptidase** | |
| 1. *thaliana* | |
| AAN41363 | A1 |
| At3g01690 | A2 |
| At5g24260 | A3 |
| NP_172818.2 | A4 |
| At5g25770 | A5 |
| At3g47560 | A6 |
| At2g24320 | A7 |
| JC8016 | A8 |
| NP_001077642.1 | A9 |
| NP_001117606.1 | A10 |
| NP_001118556.1 | A11 |
| NP_173463.1 | A12 |
| NP_173937.2 | A13 |
| NP_176862.2 | A14 |
| NP_177065.2 | A15 |
| NP_189657.1 | A16 |
| NP_193165.4 | A17 |
| NP_198470.3 | A18 |
| NP_201497.1 | A19 |
| NP_564567.1 | A20 |
| NP_568395.1 | A21 |
| NP_850473.1 | A22 |
| NP_974786.1 | A23 |
|  |  |
| *O. sativa* | |
| Os1g01830 | O1 |
| Os1g42690 | O2 |
| Os1g49510 | O3 |
| Os1g57770 | O4 |
| Os2g18850 | O5 |
| Os2g55330 | O6 |
| Os3g19410 | O7 |
| Os3g24450 | O8 |
| Os4g47360 | O9 |
| Os5g46210 | O10 |
| Os6g6770 | O11 |
| Os6g11180 | O12 |
| Os6g11190 | O13 |
| Os6g42730 | O14 |
| Os6g51410 | O15 |
| Os7g41730 | O16 |
| Os7g48970 | O17 |
| Os9g28040 | O18 |
| Os9g29950 | O19 |
| Os10g04620 | O20 |
| Os10g28020 | O21 |
| Os10g28030 | O22 |
| Os12g18860 | O23 |
|  |  |
| **Rhomboids** | |
| 1. *thaliana* | |
| AT1G52580 | RA1 |
| AT2G29050 | RA2 |
| At4g23070 | RA3 |
| At3g53780 | RA4 |
| At1g63120 | RA5 |
| At1g12750 | RA6 |
| At5g07250 | RA7 |
| At1g77860 | RA8 |
| At1g18600 | RA9 |
| At1g25290 | RA10 |
| At1g74130 | RA11 |
| At1g74140 | RA12 |
| At2g39060 | RA13 |
| At2g41160 | RA14 |
| At3g07950 | RA15 |
| At3g17611 | RA16 |
| At3g56740 | RA17 |
| At3g58460 | RA18 |
| At3g59520 | RA19 |
| At5g25752 | RA20 |
| At5g25752 | RA21 |
| *O. sativa* | |
| Os01g05430 | RO1 |
| Os04g48130 | RO2 |
| Os03g02530 | RO3 |
| Os10g37760 | RO4 |
| Os09g35730 | RO5 |
| Os08g43320 | RO6 |
| Os11g47840 | RO7 |
| Os09g28100 | RO8 |
| Os05g13370 | RO9 |
| Os01g55740 | RO10 |
| Os01g16330 | RO11 |
| Os03g44830 | RO12 |
| Os01g18100 | RO13 |
| Os01g67040 | RO14 |
| Os03g24390 | RO15 |
| Os07g46170 | RO16 |
| Os04g01300 | RO17 |
|  |  |
| **Subtilisin** | |
| 1. *thaliana* | |
| At1g01900 | SA1 |
| At1g04110 | SA2 |
| At1g20150 | SA3 |
| At1g20160 | SA4 |
| At1g30600 | SA5 |
| At1g32940 | SA6 |
| At1g32950 | SA7 |
| At1g32960 | SA8 |
| At1g32970 | SA9 |
| At1g32980 | SA10 |
| At1g62340 | SA11 |
| At1g66210 | SA12 |
| At1g66220 | SA13 |
| At2g04160 | SA14 |
| At2g05920 | SA15 |
| At2g19170 | SA16 |
| At2g39850 | SA17 |
| At3g14067 | SA18 |
| At3g14240 | SA19 |
| At3g46840 | SA20 |
| At3g46850 | SA21 |
| At4g00230 | SA22 |
| At4g10510 | SA23 |
| At4g10520 | SA24 |
| At4g10530 | SA25 |
| At4g10540 | SA26 |
| At4g10550 | SA27 |
| At4g15040 | SA28 |
| At4g20430 | SA29 |
| At4g20850 | SA30 |
| At4g21323 | SA31 |
| At4g21326 | SA32 |
| At4g21630 | SA33 |
| At4g21640 | SA34 |
| At4g21650 | SA35 |
| At4g26330 | SA36 |
| At4g30020 | SA37 |
| At4g34980 | SA38 |
| At5g03620 | SA39 |
| At5g11940 | SA40 |
| At5g19660 | SA41 |
| At5g44530 | SA42 |
| At5g45640 | SA43 |
| At5g45650 | SA44 |
| At5g51750 | SA45 |
| At5g58820 | SA46 |
| At5g58830 | SA47 |
| At5g58840 | SA48 |
| At5g59090 | SA49 |
| At5g59100 | SA50 |
| At5g59120 | SA51 |
| At5g59130 | SA52 |
| At5g59190 | SA53 |
| At5g59810 | SA54 |
| At5g67090 | SA55 |
| At5g67360 | SA56 |
|  |  |
| *O. sativa* | |
| Os01g17160 | SO1 |
| Os01g50680 | SO2 |
| Os01g52750 | SO3 |
| Os01g56320 | SO4 |
| Os01g58240 | SO5 |
| Os01g58270 | SO6 |
| Os01g58280 | SO7 |
| Os01g58290 | SO8 |
| Os01g64850 | SO9 |
| Os01g64860 | SO10 |
| Os02g10520 | SO11 |
| Os02g16940 | SO12 |
| Os02g17000 | SO13 |
| Os02g17060 | SO14 |
| Os02g17080 | SO15 |
| Os02g17090 | SO16 |
| Os02g17150 | SO17 |
| Os02g44520 | SO18 |
| Os02g44590 | SO19 |
| Os02g53850 | SO20 |
| Os02g53860 | SO21 |
| Os02g53910 | SO22 |
| Os02g53970 | SO23 |
| Os03g02750 | SO24 |
| Os03g04950 | SO25 |
| Os03g06290 | SO26 |
| Os03g13930 | SO27 |
| Os03g31630 | SO28 |
| Os03g40830 | SO29 |
| Os03g55350 | SO30 |
| Os04g02960 | SO31 |
| Os04g02980 | SO32 |
| Os04g03100 | SO33 |
| Os04g03710 | SO34 |
| Os04g03810 | SO35 |
| Os04g03850 | SO36 |
| Os04g10360 | SO37 |
| Os04g35140 | SO38 |
| Os04g45960 | SO39 |
| Os04g47150 | SO40 |
| Os04g47160 | SO41 |
| Os05g30580 | SO42 |
| Os05g36010 | SO43 |
| Os06g06810 | SO44 |
| Os06g40700 | SO45 |
| Os06g41880 | SO46 |
| Os06g48650 | SO47 |
| Os07g39020 | SO48 |
| Os07g48650 | SO49 |
| Os08g23740 | SO50 |
| Os08g35090 | SO51 |
| Os09g26920 | SO52 |
| Os09g30250 | SO53 |
| Os09g36110 | SO54 |
| Os10g25450 | SO55 |
| Os10g38080 | SO56 |
| Os11g15520 | SO57 |
| Os12g23980 | SO58 |
