# Supplementary material for: Improved Detection of Remote Homologues Using Cascade PSI-BLAST: Influence of Neighbouring Protein Families on Sequence Coverage
Source: PLoS One. 2013 Feb 20;8(2):e56449. doi: 10.1371/journal.pone.0056449 (PMC3577913; doi:10.1371/journal.pone.0056449)
Supplement: Table S2 — Precision score of POP, rhomboids and subtilisin query sequences. (DOC) [file pone.0056449.s010.doc]

| Prolyl oligopeptidase  Table S2: Precision score of POP, rhomboids and subtilisin query sequences | TP-Cascade PSI-BLAST | FP-Cascade PSI-BLAST | Precision score  Cascade PSI-BLAST |
| --- | --- | --- | --- |
| A1_AAN41363 | 4159 | 0 | 1 |
| A6_At3g47560 | 6110 | 0 | 1 |
| A5_At5g25770 | 6698 | 0 | 1 |
| A8_JC8016 | 6046 | 0 | 1 |
| A2_At3g01690 | 5894 | 0 | 1 |
| A3_At5g24260 | 6122 | 0 | 1 |
| A9_NP_001077642.1 | 7280 | 0 | 1 |
| A10_NP_001117606.1 | 6054 | 0 | 1 |
| A13_NP_173937.2 | 4146 | 0 | 1 |
| A14_NP_176862.2 | 6303 | 0 | 1 |
| A15_NP_177065.2 | 6015 | 0 | 1 |
| A17_NP_193165.4 | 6044 | 0 | 1 |
| A22_NP_850473.1 | 4730 | 0 | 1 |
| A19_NP_201497.1 | 6183 | 0 | 1 |
| A4_NP_172818.2 | 5935 | 0 | 1 |
| A12_NP_173463.1 | 6138 | 0 | 1 |
| A18_NP_198470.3 | 6125 | 0 | 1 |
| A20_NP_564567.1 | 6134 | 0 | 1 |
| A21_NP_568395.1 | 6268 | 0 | 1 |
| O23_OS_12g18860 | 5917 | 0 | 1 |
| O4_Os_01g57770 | 6640 | 0 | 1 |
| O2_Os_01g42690 | 6322 | 0 | 1 |
| O11_OS_06g6770 | 6063 | 0 | 1 |
| O6_OS_2g55330 | 6207 | 0 | 1 |
| O9_OS_04g47360 | 6029 | 0 | 1 |
| O8_OS_3g24450 | 6060 | 0 | 1 |
| O7_Os_3g19410 | 6199 | 0 | 1 |
| O12_OS_06g11180 | 6398 | 0 | 1 |
| O13_Os_06g11190 | 6047 | 0 | 1 |
| O14_Os_06g42730 | 6216 | 0 | 1 |
| O16_Os_7g41730 | 6152 | 0 | 1 |
| O18_OS_09g28040 | 7552 | 0 | 1 |
| O19_Os_09g29950 | 6089 | 0 | 1 |
| O20_OS_10g04620 | 5904 | 0 | 1 |
| O21_OS_10g28020 | 6216 | 0 | 1 |
| O5_Os_2g18850 | 6543 | 0 | 1 |
| O15_Os_6g51410 | 6083 | 0 | 1 |
| O22_Os_10g28030 | 6169 | 0 | 1 |
| Os01g18830 | 6155 | 0 | 1 |
| O10_Os_5g46210 | 6017 | 0 | 1 |
| O3_OS1g49510 | 5939 | 0 | 1 |
|  |  |  |  |
| Rhomboids |  |  |  |
| AT1G52580 | 203 | 0 | 1 |
| AT2G29050 | 203 | 0 | 1 |
| At4g23070 | 203 | 0 | 1 |
| At3g53780 | 203 | 0 | 1 |
| At1g63120 | 203 | 0 | 1 |
| At1g12750 | 203 | 0 | 1 |
| At5g07250 | 203 | 0 | 1 |
| At1g77860 | 203 | 0 | 1 |
| At1g18600 | 203 | 0 | 1 |
| At1g25290 | 203 | 0 | 1 |
| At1g74130 | 203 | 0 | 1 |
| At1g74140 | 203 | 0 | 1 |
| At2g39060 | 1 | 0 | 1 |
| At2g41160 | 3 | 0 | 1 |
| At3g07950 | 203 | 0 | 1 |
| At3g17611 | 203 | 0 | 1 |
| At3g56740 | 3 | 0 | 1 |
| At3g58460 | 204 | 0 | 1 |
| At3g59520 | 203 | 0 | 1 |
| At5g25752 | 203 | 0 | 1 |
| At5g38510 | 203 | 0 | 1 |
| LOC_Os01g05430 | 202 | 0 | 1 |
| LOC_Os04g48130 | 202 | 0 | 1 |
| LOC_Os03g02530 | 203 | 0 | 1 |
| LOC_Os10g37760 | 203 | 0 | 1 |
| LOC_Os09g35730 | 203 | 0 | 1 |
| LOC_Os08g43320 | 203 | 0 | 1 |
| LOC_Os11g47840 | 203 | 0 | 1 |
| LOC_Os09g28100 | 203 | 0 | 1 |
| LOC_Os05g13370 | 203 | 0 | 1 |
| LOC_Os01g55740 | 203 | 0 | 1 |
| LOC_Os01g16330 | 3 | 0 | 1 |
| LOC_Os03g44830 | 202 | 0 | 1 |
| LOC_Os01g18100 | 203 | 0 | 1 |
| LOC_Os01g67040 | 203 | 0 | 1 |
| LOC_Os03g24390 | 203 | 0 | 1 |
| LOC_Os07g46170 | 203 | 0 | 1 |
| LOC_Os04g01300 | 203 | 0 | 1 |
|  |  |  |  |
| Subtilisins |  |  |  |
| At1g01900 | 844 | 442 | 65.62986003 |
| At1g04110 | 835 | 304 | 73.30992098 |
| At1g20150 | 848 | 538 | 61.18326118 |
| At1g20160 | 805 | 362 | 68.98029135 |
| At1g30600 | 836 | 363 | 69.72477064 |
| At1g32940 | 844 | 233 | 78.36583101 |
| At1g32950 | 848 | 555 | 60.44191019 |
| At1g32960 | 763 | 110 | 87.3997709 |
| At1g32970 | 763 | 110 | 87.3997709 |
| At1g32980 | 845 | 404 | 67.6541233 |
| At1g62340 | 842 | 322 | 72.33676976 |
| At1g66210 | 850 | 367 | 69.84387839 |
| At1g66220 | 850 | 432 | 66.30265211 |
| At2g04160 | 847 | 280 | 75.1552795 |
| At2g05920 | 850 | 114 | 88.17427386 |
| At2g19170 | 840 | 262 | 76.22504537 |
| At2g39850 | 850 | 608 | 58.29903978 |
| At3g14067 | 840 | 471 | 64.07322654 |
| At3g14240 | 850 | 602 | 58.5399449 |
| At3g46840 | 850 | 463 | 64.73724296 |
| t3g46850 | 849 | 700 | 54.80955455 |
| At4g00230 | 850 | 573 | 59.73295854 |
| At4g10510 | 850 | 115 | 88.08290155 |
| At4g10520 | 847 | 451 | 65.25423729 |
| At4g10530 | 847 | 451 | 65.25423729 |
| At4g10540 | 850 | 274 | 75.6227758 |
| At4g10550 | 850 | 266 | 76.16487455 |
| At4g15040 | 850 | 246 | 77.55474453 |
| At4g20430 | 845 | 503 | 62.68545994 |
| At4g20850 | 842 | 675 | 55.50428477 |
| At4g21323 | 838 | 667 | 55.68106312 |
| At4g21326 | 844 | 796 | 51.46341463 |
| At4g21630 | 840 | 320 | 72.4137931 |
| At4g21640 | 838 | 730 | 53.44387755 |
| At4g21650 | 841 | 617 | 57.68175583 |
| At4g26330 | 786 | 437 | 64.26819297 |
| At4g30020 | 761 | 111 | 87.2706422 |
| At4g34980 | 849 | 355 | 70.51495017 |
| At5g03620 | 842 | 315 | 72.77441659 |
| At5g11940 | 850 | 138 | 86.03238866 |
| At5g19660 | 842 | 161 | 83.94815553 |
| At5g44530 | 850 | 189 | 81.80943215 |
| At5g45650 | 839 | 208 | 80.13371538 |
| At5g51750 | 844 | 453 | 65.07324595 |
| At5g58820 | 850 | 694 | 55.05181347 |
| At5g58830 | 841 | 545 | 60.67821068 |
| At5g58840 | 842 | 128 | 86.80412371 |
| At5g59090 | 849 | 412 | 67.32751784 |
| At5g59100 | 840 | 620 | 57.53424658 |
| At5g59120 | 848 | 397 | 68.1124498 |
| At5g59130 | 843 | 609 | 58.05785124 |
| At5g59190 | 845 | 319 | 72.59450172 |
| At5g59810 | 839 | 542 | 60.75307748 |
| At5g67090 | 848 | 412 | 67.3015873 |
| At5g67360 | 850 | 743 | 53.35844319 |
| Os01g50680 | 850 | 113 | 88.26583593 |
| Os01g52750 | 839 | 459 | 64.63790447 |
| Os01g56320 | 840 | 508 | 62.31454006 |
| Os01g58240 | 839 | 493 | 62.98798799 |
| Os01g58270 | 848 | 579 | 59.4253679 |
| Os01g58290 | 847 | 442 | 65.7098526 |
| Os01g64850 | 837 | 807 | 50.91240876 |
| Os01g64860 | 845 | 290 | 74.44933921 |
| Os02g10520 | 748 | 986 | 43.1372549 |
| Os02g16940 | 850 | 295 | 74.23580786 |
| Os02g17000 | 850 | 113 | 88.26583593 |
| Os02g17060 | 847 | 235 | 78.28096118 |
| Os02g17080 | 850 | 112 | 88.35758836 |
| Os02g17090 | 849 | 413 | 67.27416799 |
| Os02g17150 | 849 | 364 | 69.99175598 |
| Os02g44520 | 842 | 337 | 71.41645462 |
| Os02g44590 | 841 | 819 | 50.6626506 |
| Os02g53850 | 848 | 130 | 86.70756646 |
| Os02g53860 | 843 | 946 | 47.12129681 |
| Os02g53910 | 841 | 725 | 53.7037037 |
| Os02g53970 | 841 | 580 | 59.18367347 |
| Os03g02750 | 848 | 326 | 72.23168654 |
| Os03g04950 | 849 | 625 | 57.59837178 |
| Os03g06290 | 850 | 422 | 66.82389937 |
| Os03g31630 | 835 | 595 | 58.39160839 |
| Os03g40830 | 841 | 401 | 67.71336554 |
| Os04g02960 | 850 | 229 | 78.77664504 |
| Os04g02980 | 849 | 416 | 67.11462451 |
| Os04g03100 | 850 | 444 | 65.6877898 |
| Os04g03710 | 850 | 904 | 48.46066135 |
| Os04g03810 | 842 | 482 | 63.59516616 |
| Os04g03850 | 847 | 553 | 60.5 |
| Os04g35140 | 850 | 798 | 51.5776699 |
| Os04g47150 | 842 | 348 | 70.75630252 |
| Os04g47160 | 806 | 174 | 82.24489796 |
| Os05g30580 | 845 | 112 | 88.29676071 |
| Os05g36010 | 843 | 239 | 77.91127542 |
| Os06g06810 | 835 | 315 | 72.60869565 |
| Os06g40700 | 836 | 109 | 88.46560847 |
| Os06g48650 | 842 | 568 | 59.71631206 |
| Os07g39020 | 848 | 127 | 86.97435897 |
| Os07g48650 | 845 | 574 | 59.54897815 |
| Os08g23740 | 840 | 580 | 59.15492958 |
| Os08g35090 | 839 | 205 | 80.36398467 |
| Os09g26920 | 839 | 719 | 53.85109114 |
| Os09g30250 | 835 | 245 | 77.31481481 |
| Os09g36110 | 840 | 314 | 72.79029463 |
| Os10g25450 | 844 | 493 | 63.12640239 |
| Os10g38080 | 834 | 945 | 46.88026981 |
| Os11g15520 | 850 | 138 | 86.03238866 |
| Os12g23980 | 841 | 499 | 62.76119403 |
| **Average precision** |  |  | **0.79** |

TP: true positive, FP: false positive
