# Supplementary material for: Improved Detection of Remote Homologues Using Cascade PSI-BLAST: Influence of Neighbouring Protein Families on Sequence Coverage
Source: PLoS One. 2013 Feb 20;8(2):e56449. doi: 10.1371/journal.pone.0056449 (PMC3577913; doi:10.1371/journal.pone.0056449)
Supplement: Table S5 — Jackknifing of the α/β hydrolase superfamily using POP as a query sequence representing coverage at the fold level. (DOC) [file pone.0056449.s013.doc]

Table S5: Jackknifing of the α/β hydrolase superfamily using POP as a query sequence representing coverage at the fold level.

| Query | **Precision score- plant POP family level** | | **Coverage- fold level** | | **Precision score- Fold level** | |
| --- | --- | --- | --- | --- | --- | --- |
|  | **PSI-BLAST** | **Cascade PSI-BLAST** | PSI-BLAST | Cascade PSI-BLAST | **PSI-BLAST** | **Cascade PSI-BLAST** |
| POP(At3g47560) | 0.1 | 0.66 | 20.96 | 64.12 | 100 | 100 |
| >1.1 (Acetylcholinesterase) | 0.05 | 0.52 | 20.98 | 59.48 | 100 | 100 |
| >1.11(Epoxide_hydrolase) | 0.1 | 0.54 | 20.96 | 61.35 | 100 | 100 |
| >1.12 (Haloperoxidase) | 0.1 | 0.43 | 20.96 | 61.99 | 100 | 100 |
| >1.13(Thioesterases) | 0.1 | 0.45 | 20.96 | 61.75 | 100 | 100 |
| >1.14(Carboxylesterase/Thioesterase) | 0.15 | 0.57 | 20.91 | 61.62 | 100 | 100 |
| >1.15 (A_novel_bacterial_esterase) | 0.1 | 0.56 | 20.92 | 63.27 | 100 | 100 |
| >1.17 (Fungal-lipase) | 0.1 | 0.55 | 20.96 | 61.77 | 100 | 100 |
| >1.18 (Bacterial_Lipase) | 0.1 | 0.56 | 20.96 | 62.91 | 100 | 100 |
| >1.19 (Pancreatic_lipase) | 0.15 | 0.45 | 20.91 | 62.14 | 100 | 100 |
| 1.2 (Carboxylesterase) | 0.05 | 0.57 | 20.98 | 59.47 | 100 | 100 |
| >1.21 (PepX_cataltic_domain_like) | 0.1 | 0.52 | 20.96 | 61.69 | 100 | 100 |
| >1.22 (Thioesterase-domain-of-polypeptide) | 0.15 | 0.56 | 20.91 | 62.95 | 100 | 100 |
| >1.23 (Ccg1_TafII250-interacting_factor) | 0.1 | 0.50 | 20.96 | 63.78 | 100 | 100 |
| >1.25 (Acetyl_xylan_esterse_like) | 0.15 | 0.59 | 20.93 | 65.37 | 100 | 100 |
| >1.26 (Biotin_biosynthesis_protein) | 0.15 | 0.56 | 20.95 | 62.81 | 100 | 100 |
| >1.3(Mycobacterial_Antigen) | 0.05 | 0.49 | 20.83 | 60.94 | 100 | 100 |
| >1.32 (Putative_serine_hydrolase_Ydr428) | 0.1 | 0.48 | 20.95 | 62.45 | 100 | 100 |
| >1.36 (Atu1826_like_XC6422) | 0.15 | 0.56 | 20.91 | 62.95 | 100 | 100 |
| >1.6 (Gastric_lipase) | 0.15 | 0.48 | 20.91 | 62.14 | 100 | 100 |
| >1.16(Lipase) | 0.1 | 0.52 | 20.96 | 61.32 | 100 | 100 |
| >1.24 (Dipeptidyl_peptidase) | 0.1 | 0.57 | 20.95 | 61.99 | 100 | 100 |
| >1.30(Cutinase) | 0.15 | 0.56 | 20.91 | 63.05 | 100 | 100 |
| >1.33(Acylamino-acid-releasing_enzyme) | 0.15 | 0.60 | 20.86 | 65.29 | 100 | 100 |
| >1.37(Polyhydroxybutyrate_depolymerase) | 0.15 | 0.54 | 20.91 | 62.96 | 100 | 100 |
| >1.40(O_acetyltransferase-) | 0.15 | 0.58 | 20.91 | 60.54 | 100 | 100 |
| >1.27 (Hypothetical_protein_TT1662) | 0.05 | 0.40 | 20.98 | 61.26 | 100 | 100 |
| >1.28(Aclacinomycin_methylesterase) | 0.1 | 0.51 | 20.70 | 62.97 | 100 | 100 |
| >1.34(Hypothetical_esterase_YJL068C) | 0.05 | 0.39 | 20.80 | 61.49 | 100 | 100 |
| >1.35(Hypothetical_protein_VC1974) | 0.15 | 0.44 | 20.74 | 61.87 | 100 | 100 |
| >1.38(IroE_like) | 0.05 | 0.45 | 20.77 | 61.71 | 100 | 100 |
| >1.39(TTHA1544) | 0.1 | 0.42 | 20.74 | 51.91 | 100 | 100 |
| >1.41(2_2_6_hydropseudooxynicotine_hydrolase) | 0.05 | 0.53 | 20.49 | 63.41 | 100 | 100 |
| >1.5(Serine_carboxypeptidase) | 0.1 | 0.44 | 20.74 | 62.52 | 100 | 100 |
| >1.7(Proline_iminopeptidase) | 0.1 | 0.36 | 20.43 | 63.05 | 100 | 100 |
| >1.8(Haloalkane_dehalogenase) | 0.15 | 0.46 | 20.66 | 61.98 | 100 | 100 |
| >1.9(Dienelactone_hydrolase) | 0.1 | 0.44 | 20.75 | 60.32 | 100 | 100 |
| >1.10(Carbon-carbon bond hydrolase) | 0.15 | 0.54 | 20.45 | 62.64 | 100 | 100 |
| >1.20(Hydroxynitrile_lyase) | 0.15 | 0.43 | 20.59 | 63.24 | 100 | 100 |
| >1.31(YdeN) | 0.1 | 0.37 | 20.74 | 62.33 | 100 | 100 |

>indicates removed family
